# Evaluation of Mechanisms to Improve Performance of Mobile Phone Surveys in Low- and Middle-Income Countries: Research Protocol

**DOI:** 10.2196/resprot.7534

**Published:** 2017-05-05

**Authors:** Dustin G Gibson, George William Pariyo, Adaeze C Wosu, Abigail R Greenleaf, Joseph Ali, Saifuddin Ahmed, Alain B Labrique, Khaleda Islam, Honorati Masanja, Elizeus Rutebemberwa, Adnan A Hyder

**Affiliations:** ^1^ Department of International Health Johns Hopkins Bloomberg School of Public Health Baltimore, MD United States; ^2^ Department of Epidemiology Johns Hopkins Bloomberg School of Public Health Baltimore, MD United States; ^3^ Department of Population, Family and Reproductive Health Johns Hopkins Bloomberg School of Public Health Baltimore, MD United States; ^4^ Berman Institute of Bioethics Johns Hopkins University Baltimore, MD United States; ^5^ Institute of Epidemiology, Disease Control and Research Dhaka Bangladesh; ^6^ Ifakara Health Institute Dar es Salaam United Republic Of Tanzania; ^7^ Makerere University School of Public Health Makerere University College of Health Science Kampala Uganda

**Keywords:** IVR, CATI, Bangladesh, Tanzania, Uganda, mHealth, mobile phone survey, noncommunicable diseases, survey methodology

## Abstract

**Background:**

Mobile phone ownership and access have increased rapidly across low- and middle-income countries (LMICs) within the last decade. Concomitantly, LMICs are experiencing demographic and epidemiologic transitions, where non-communicable diseases (NCDs) are increasingly becoming leading causes of morbidity and mortality. Mobile phone surveys could aid data collection for prevention and control of these NCDs but limited evidence of their feasibility exists.

**Objective:**

The objective of this paper is to describe a series of sub-studies aimed at optimizing the delivery of interactive voice response (IVR) and computer-assisted telephone interviews (CATI) for NCD risk factor data collection in LMICs. These sub-studies are designed to assess the effect of factors such as airtime incentive timing, amount, and structure, survey introduction characteristics, different sampling frames, and survey modality on key survey metrics, such as survey response, completion, and attrition rates.

**Methods:**

In a series of sub-studies, participants will be randomly assigned to receive different airtime incentive amounts (eg, 10 minutes of airtime versus 20 minutes of airtime), different incentive delivery timings (airtime delivered before survey begins versus delivery upon completion of survey), different survey introductions (informational versus motivational), different narrative voices (male versus female), and different sampling frames (random digit dialing versus mobile network operator-provided numbers) to examine which study arms will yield the highest response and completion rates. Furthermore, response and completion rates and the inter-modal reliability of the IVR and CATI delivery methods will be compared.

**Results:**

Research activities are expected to be completed in Bangladesh, Tanzania, and Uganda in 2017.

**Conclusions:**

This is one of the first studies to examine the feasibility of using IVR and CATI for systematic collection of NCD risk factor information in LMICs. Our findings will inform the future design and implementation of mobile phone surveys in LMICs.

## Introduction

Effective prevention and control of non-communicable diseases (NCDs) centers on surveillance and reduction of exposure to risk factors that give rise to these conditions. Four behavioral, and largely modifiable, risk factors account for the majority of NCDs: unhealthy diet, tobacco use, inadequate physical activity, and excessive use of alcohol [1]. Surveillance provides crucial information on disease burden to guide resource planning and allocation and evaluation of public health interventions and programs [2]. Unfortunately, many low- and middle-income countries (LMICs) face the challenge of implementing effective and systematic ways of tracking NCD risk factors and collecting timely and high-quality data. The rise of mobile phones in LMICs presents an opportunity to use this technology to aid NCD risk factor data collection. Currently, worldwide mobile phone subscriptions have reached 99.7 subscriptions per 100 persons, with 94.1 subscriptions per 100 persons in LMICs [3].

High levels of mobile phone access and ownership afford new opportunities to conduct surveys. As a complement to household surveys, respondents can be interviewed over their personal mobile phones. The most common types of mobile phone surveys (MPSs) are short message service (SMS), computer-assisted telephone interview (CATI), and interactive voice response (IVR). In CATI, an interviewer questions the respondent over the phone. With SMS, brief text messages are used to communicate between devices [4]. In IVR, users interact with a database programmed with questions and a series of pre-recorded answers to the questions, linked to a specific numeric key or numeric response on a touch-tone phone keypad (eg, “Press 1 if you are male, press 3 if you are female”).

The use of MPSs to collect population health estimates in LMICs is in its early stages [5]. Similarly, there is little evidence on the usability and reliability of MPSs and mechanisms to improve survey response, completion and representativeness in LMIC settings. Through a series of 7 sub-studies, this protocol seeks to address the following specific objectives: (1) evaluate the impact of incentive amount, timing, and structure on response, completion, and refusal rates of an IVR-administered NCD risk factor survey; (2) assess the effect of different survey introduction content (eg, informational versus motivational introduction) and modality on response, completion, and refusal rates of an IVR-administered NCD risk factor survey; (3) examine the differences in survey metrics, such as representativeness, completeness, and response rate between different sampling frames of mobile phone numbers; and (4) evaluate the differences in response, completion, and refusal rates between IVR and CATI surveys and establish the inter-modal reliability.

The research studies described in this protocol are part of the NCD research and development arm of the Bloomberg Data for Health Initiative (BD4HI). The BD4HI is an effort led by Bloomberg Philanthropies to improve health by improving the quality and availability of public health data across the globe. The motivation, key goals, partners, and components of this initiative have been described in another paper by our research team [6]. Briefly, the BD4HI NCD research and development agenda includes assessing ways to optimize MPS for NCD risk factor data collection in LMICs.

## Methods

### Formative Phase

Prior to the implementation of the sub-studies, a number of formative activities will be conducted to contextualize the use of MPSs for optimal performance in each country. Briefly, a 3-pronged, standardized approach is used to inform the design and adapt a centrally developed NCD questionnaire for IVR delivery in each country. This includes key informant interviews (KII) with personnel (eg, in government, regulatory agencies, and academic experts) who have in-depth knowledge of relevance to NCDs or the conduct of mobile phone surveys. Additionally, we will conduct focus group discussions (FGDs) to explore community perceptions and willingness to participate in a MPS and identify potential challenges, barriers, and solutions. Finally, user-group testing will be carried out where community members complete an IVR survey in the presence of study staff. These formative activities will be used to create a revised questionnaire containing appropriate local examples, terminologies, and measurements. The country-adapted questionnaire and response options will be used in each of the 7 sub-studies.

### Evaluation Phase

#### Setting and Participants

The research activities will be implemented in Bangladesh, Tanzania, and Uganda. Information on collaborating partners, mobile phone subscription rates [7], mobile network operators (MNOs), and survey languages is summarized in Table 1. The term “subscription” in Table 1, and throughout this protocol, refers to different forms of mobile phone ownership including monthly subscriptions and pre-payment options which are common in LMICs.

**Table 1:** Country partners, survey languages, and mobile phone subscriptions in each country.

Country	Mobile phone subscriptions per 100 people, n	Mobile network operators in country, n	Implementing partner	Languages for mobile phone survey
Bangladesh	83	5	Institute of Epidemiology, Disease Control and Research (IEDCR)	Bangla, English
Tanzania	76	5	Ifakara Health Institute (IHI)	Kiswahili, English
Uganda	50	6	Makerere University School of Public Health (MakSPH)	Luganda, Runyakitara, Luo, English

In each of the 3 countries, participants for the MPS, except for sub-study 6, will be selected through random digit dialing (RDD) [8]. For each country, we will identify all active MNOs and their respective prefixes that lead a 10-digit mobile phone number. Using these unique prefixes, the remaining digits will then be randomly generated via a computer to create a random sample of mobile phone numbers to which the surveys will be delivered (Table 2). Participants that are selected using RDD will be asked to provide consent and their age. Participants who do not indicate their age or report being less than 18 years old will be excluded from the study.

#### Study Design

The 7 sub-studies that evaluate mechanisms to improve survey response and cooperation rates of an IVR-administered NCD risk factor survey or compare differences in survey performance between IVR and CATI surveys will be conducted in the 3 countries. The description of each sub-study and a summary of their activities are provided in Table 3. The outcome for every sub-study will be contact, response, completion and refusal rates, and demographic representativeness. In addition, sub-study 7 will include the inter-modal reliability between IVR and CATI-administered surveys.

**Table 2:** Generating a sample through random digit dialing.

Mobile network operator	Unique prefixes^a^	Examples of RDD^b^ phone numbers^c^
A		
	0772-XXX-XXX	0772-111-222, 0772-111-222
	0773-XXX-XXX	0773-234-567, 0773-234-567
	0774-XXX-XXX	0774-743-128, 0774-743-128
B		
	0791-XXX-XXX	0791-381-123, 0791-237-268
	0792-XXX-XXX	0792-326-888, 0792-666-418
C		
	0745-XXX-XXX	0745-674-190, 0745-552-383
	0746-XXX-XXX	0746-901-643, 0746-434-122
	0720-XXX-XXX	0720-023-528, 0720-712-090
	0721-XXX-XXX	0721-057-444, 0721-723-889

^a^Following the unique prefixes, the remaining digits (“X”) are randomly generated using a computer to produce a 10-digit phone number.

^b^RDD: random digit dialing.

^c^The telephone numbers that appear in this table were randomly generated and any similarity with an actual subscriber’s number is purely coincidental.

**Table 3:** Summary description of the sub-studies.

Sub-study	Name	Study arms	Sampling	Country
1	Incentive amount	No airtime	RDD^a^	Bangladesh, Uganda
		X airtime post-IVR^b^		
		2X airtime post-IVR		
2	Incentive timing	No airtime	RDD	Bangladesh, Uganda
		Approximately 20% airtime pre-IVR; approximately 80% airtime post		
		100% airtime post-IVR		
3	Incentive structure	No airtime	RDD	Bangladesh, Uganda
		Fixed X airtime post-IVR		
		Lottery airtime of at least 5X post-IVR		
4	Introduction phrasing and voice	Male voice, informational introduction	RDD	Bangladesh, Uganda
		Female voice, informational introduction		
		Male voice, motivational introduction		
		Female Voice, motivational introduction		
5	Introduction modality	IVR introduction, IVR survey	RDD	Bangladesh, Tanzania
		CATI^c^ introduction, IVR survey		
6	Sampling frame	IVR survey	MNO^d^-provided numbers	Bangladesh
7	Survey modality	CATI survey first, IVR survey 7 days later	RDD	Bangladesh, Tanzania
		IVR survey first, CATI survey 7 days later		

^a^RDD: random digit dialing.

^b^IVR: interactive voice response.

^c^CATI: computer-assisted telephone interview.

^d^MNO: mobile network operator.

#### Sub-Study 1: Incentive Amount

This sub-study evaluates whether there is a differential effect in completion rates of an IVR-administered survey based on the incentive amount. Participants will be randomized to receive 1 of 3 incentive amounts, in the form of airtime, which will be sent to participants upon completion of the IVR survey. The study arms are (1) no incentive; (2) X US dollar airtime incentive, where X is commensurate with the expected time commitment as a function of daily wages (the exact amount will be guided by FGDs and in consultation with country partners); and (3) 2X airtime incentive transferred after survey completion (where 2X is twice the amount of the incentive in arm 2). This sub-study will be conducted in Bangladesh and Uganda.

#### Sub-Study 2: Incentive Timing

This sub-study examines whether the timing of incentive delivery (either pre-survey or post-survey) has an effect on survey response and completion rates of an IVR-administered survey. Participants will be randomized to 1 of the following 3 study arms: (1) no incentive; (2) an airtime incentive sent before the survey, where 10% to 40% of the total incentive (X) is sent to the respondent’s mobile phone prior to initiation of the survey, and with the remaining 60% to 90% being sent after all questions answered; or (3) a promised incentive where 100% of the incentive amount is sent to the respondent’s mobile phone only after the IVR survey is completed. This sub-study will be conducted in Bangladesh and Uganda.

#### Sub-Study 3: Incentive Structure

This sub-study evaluates whether the incentive structure (a fixed incentive amount or a lottery-based incentive) has an effect on completion rate of an IVR-administered survey. Respondents will be randomized to 1 of the following 3 study arms: (1) no incentive; (2) a fixed airtime incentive delivered after survey completion (where the amount of the incentive is guided by sub-study 1); or (3) a lottery airtime incentive of an amount at least 5 times greater than the amount in the second study arm, where the odds of winning the lottery are 1 to 20 (the lottery incentive will be delivered upon completion of the survey and the precise odds for winning will be guided by formative work conducted in each country). This sub-study will be conducted in Bangladesh and Uganda.

#### Sub-Study 4: Introduction Phrasing and Survey Voice

This sub-study assesses whether the content of the IVR survey’s introduction (motivational versus informational) and the survey’s narrative voice (male versus female) has an effect on contact, response, and/or completion rates. Participants will be randomized to 1 of 4 study arms with varying survey introduction content and voices: (1) male survey narrator with an informational survey introduction; (2) male survey narrator with a motivational survey introduction; (3) female survey narrator with an informational survey introduction; or (4) female survey narrator with a motivational survey introduction. The content of the survey’s motivational and informational introductions will be guided by formative work conducted in each country. Participants in each study arm will receive the same airtime incentive. This sub-study will be conducted in Bangladesh and Uganda.

#### Sub-Study 5: Survey Introduction Modality

This sub-study evaluates the effect of the survey introduction’s modality (human operator versus IVR introduction) on survey completion rates. Participants will be randomized to 1 of 2 arms: (1) a CATI-administered introduction with an IVR survey or (2) a full IVR survey. In the first arm, a human will call the RDD-generated mobile phone number, read the survey’s introduction, and the operator will answer any questions the participant may have. Respondents that agree to participate will then be sent an IVR survey. In the second arm, participants will not receive this human introduction. Participants in each study arm will receive the same airtime incentive. This sub-study will be conducted in Bangladesh and Tanzania.

#### Sub-Study 6: Sampling Frame

This sub-study examines whether the source of the mobile phone numbers has a differential effect on IVR survey response and completion rates. The previous sub-studies and sub-Study 7 rely on an RDD sampling frame. In this sub-study, we will obtain a de-identified list of mobile phone numbers from a MNO where participants have consented to be contacted for additional studies. This sub-study will be conducted in Bangladesh.

#### Sub-Study 7: Survey Modality

**Figure 1:**
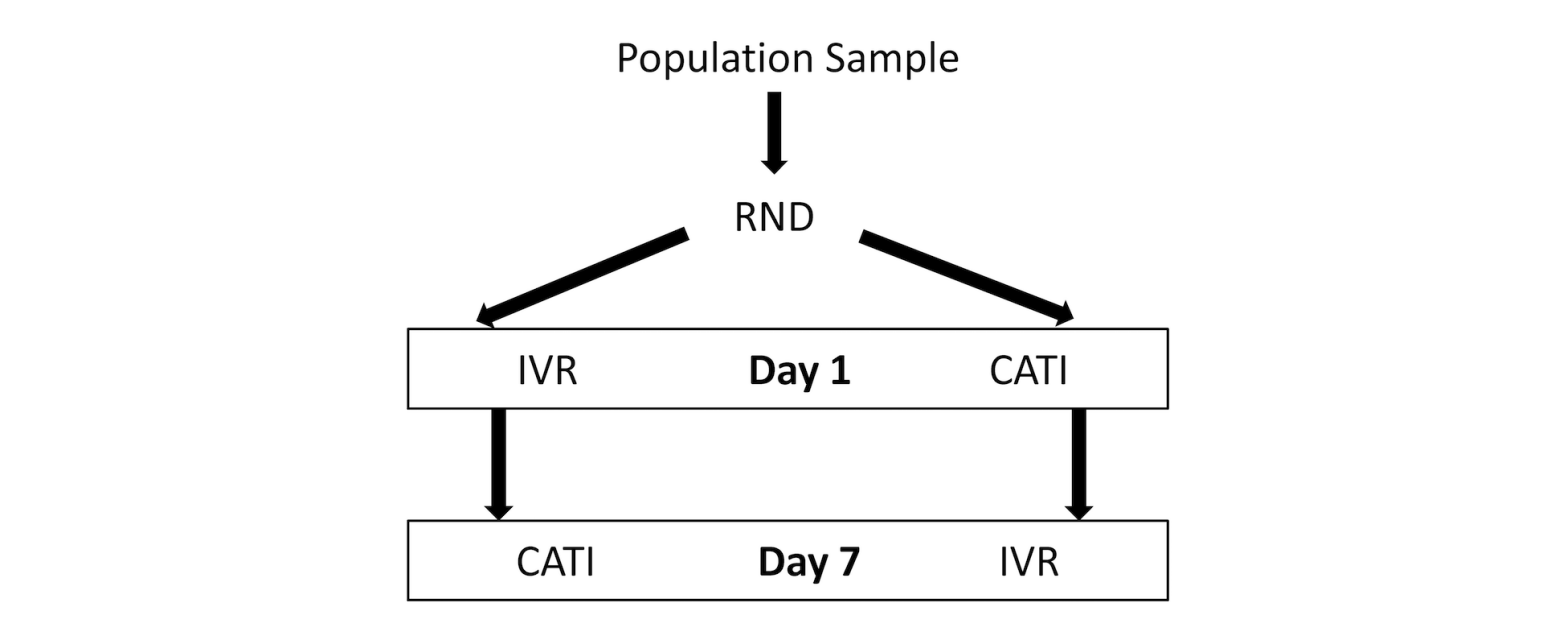
Study design for sub-study 7.

This sub-study evaluates the effect of the survey modality (IVR versus CATI) on the survey’s response and completion rates and assesses the inter-modal reliability. Participants will be randomized to 1 of 2 arms: (1) IVR then CATI; or (2) CATI then IVR. Participants in the first study arm will receive an IVR survey first, followed by a CATI survey 7 days later (Figure 1). Participants in the second study arm will receive a CATI survey first followed by an IVR survey 7 days later. At initial enrollment, it will be clearly explained to participants that they are being enrolled in a study where they will be contacted twice. The questionnaires used in both study arms will be the same. This crossover design will allow for an assessment of response consistency, adjusted for the risk of “priming” after exposure to the prior modality. This is the only sub-study that will require participants to answer the NCD survey twice. The amount, timing, and structure of incentive will be guided by the first 3 sub-studies where the incentive that yielded the highest response and completion rates will be provided to all participants in sub-study 7. This sub-study will be conducted in Bangladesh and Tanzania.

#### Follow-Up of Study Participants

In order to better understand reasons for refusal and survey attrition, to assess the usability of the IVR platform, and to receive feedback on the incentives and survey introductions, we will call a sub-sample of participants from each sub-study and administer a short MPS. For each of these sub-studies, we will call back a random sample of 30 individuals who (1) were age-eligible and consented but did not complete the survey; (2) refused to consent to the survey; and (3) were identified as non-responders (ie, listened to a portion of the survey introduction but disconnected from the survey).

### Questionnaire

Experts in NCDs, mobile health, and survey methodology convened in 2015 to develop a NCD risk factor questionnaire that could be adapted to a MPS. Questions were selected from standardized surveys such as World Health Organization (WHO) STEPwise Surveillance and Tobacco Questions for Surveys [9,10]. Question selection was guided by the Global Monitoring Framework for NCDs, which included the main behavioral NCD risk factors (physical activity, alcohol consumption, tobacco use, and diet) [11]. Questions were selected independent of their perceived adaptability to an IVR platform.

**Figure 2:**
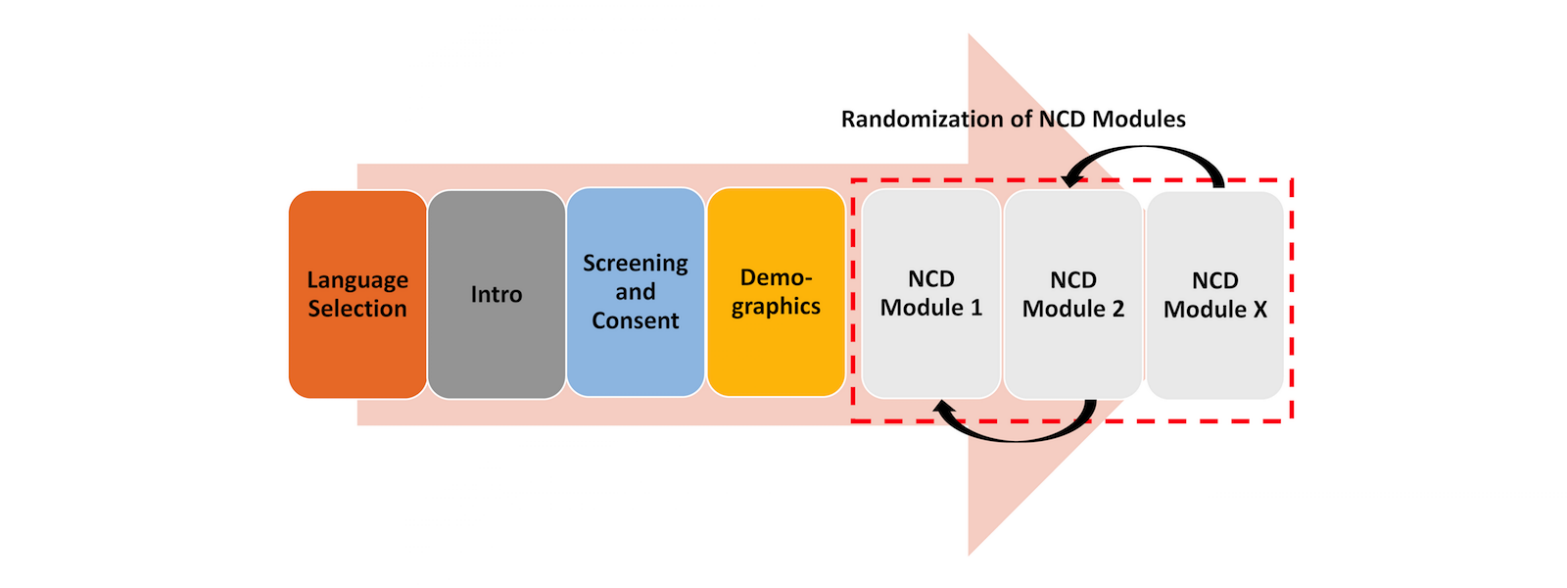
Sequence of the mobile phone survey.

The questionnaire is comprised of the following parts: (1) language selection, (2) introduction, (3) age screening, (4) consent, (5) demographic questions, (6) NCD modules, and (7) airtime delivery, as applicable (Figure 2). NCD modules are groups of topically similar NCD risk factor questions (eg, tobacco, alcohol, diet, physical activity, etc). The order of the modules will be randomly assigned. For example, one participant may be presented with questions on diet first, followed by tobacco, then alcohol, and finishing with physical activity; another respondent may be presented alcohol questions first, and then diet, then physical activity, and finally tobacco. However, the questions within each module will not be randomized.

The questionnaire will be translated into local languages with input from members of local collaborators and communities to help ensure appropriate syntax and semantics and then back-translated to ensure accuracy. After the questionnaire’s translation has been deemed acceptable, country residents fluent in the survey languages will narrate and audio record the survey. The audio recording of the survey will be uploaded into VOTOmobile’s IVR platform. VOTOmobile is a Ghana-based organization that works to develop mobile phone survey systems.

### Survey Delivery and Data Collection

Unless otherwise indicated, the logistics and timing of MPS delivery will be similar for all sub-studies. When a randomly dialed number is connected (ie, a respondent picks up the phone), the respondent will be presented with a brief pre-recorded introduction to the study and provided an opportunity to select the survey’s language. For IVR sub-studies, respondents will select a language by entering their response on their mobile phone keypad (eg, “If you would like to listen in English, press 1”).

After the language selection, the speaker will provide a more detailed description of the study that includes the purpose, duration, risks, benefits, sponsoring agency, and requirements to receive an incentive. For sub-study 4, participants in the motivational introduction arm will receive added sentences and language expressions that may further motivate them to participate in the survey. After hearing the survey’s introduction, participants will be screened for eligibility based on their age; those who indicate being less than 18 years old will be thanked for their interest and the survey will be terminated. Participants will then be asked to press a number on their keypad to indicate their willingness to continue (eg, “Press 1 if you would like to participate in this survey, press 3 if you do not want to participate in the survey”). Respondents will be asked a series of demographic questions followed by NCD modules whose order of presentation is randomized as described above.

Surveys will only be sent to participants once. Participants who were ineligible due to age, refused consent, or opted out during the survey will not have an additional survey sent. The MPS will be delivered at random times ranging from 8:00 AM to 8:00 PM local time. The exact time window to deliver the MPS will be determined by each country during the formative phase.

### Proposed Sample Sizes

Assuming a baseline survey completion percentage of 30%, in order to detect an absolute 10% difference in survey completion between two study arms at an alpha of .05 and power of 80%, it is calculated that 376 individuals who have completed an IVR survey will be needed in each study arm for each sub-study. With a completion percentage of 30%, we calculated that 1254 participants would be required to consent to the survey per study arm in each country. With 16 study arms total in these 6 sub-studies, 20,064 participants will be enrolled in each country (Table 4). We have not inflated the sample size for multiple comparisons as per the recommendation by Rothman [12]. We chose a 10% difference between study arms as our preliminary analysis indicated that a 1 dollar incentive becomes cost-neutral at this cut-off point.

**Table 4:** Number of participants needed to complete a mobile phone survey per country.

Sub-study	Study arms	Participants who completed survey per study arm, n	Total participants who completed the survey, n
Sub-study 1	3	376	1128
Sub-study 2	3	376	1128
Sub-study 3	3	376	1128
Sub-study 4	4	376	1504
Sub-study 5	2	376	752
Sub-study 6	1	376	376
Sub-study 7^a^	2	405	810
Total			6826

^a^For sub-study 7, we adjust for a 20% loss to follow-up from the first and second mobile phone survey.

For sub-study 7, the sample size is calculated based on the kappa statistic [13]. With assumption of kappa .75, a margin of error of 0.05%, an alpha of .05, and the proportion of positive responses of 0.3, 405 participants who have completed the survey per study arm are needed. Adjusting for a 20% loss to follow-up from the first and second mobile phone survey and a 30% baseline completion percentage, 1688 participants who consented will be enrolled per arm, for a total of 3376 participants across the two arms for each country.

### Data Management and Analysis

The main outcomes in this study are contact, response, refusal, and completion proportions. Our outcomes are defined based on standard definitions from the 9th edition of the American Association for Public Opinion Research (AAPOR) [14], with some modifications to accommodate our RDD MPS design. Eligible individuals will be defined as persons who confirm that they are at least 18 years old.

**Figure 3:**
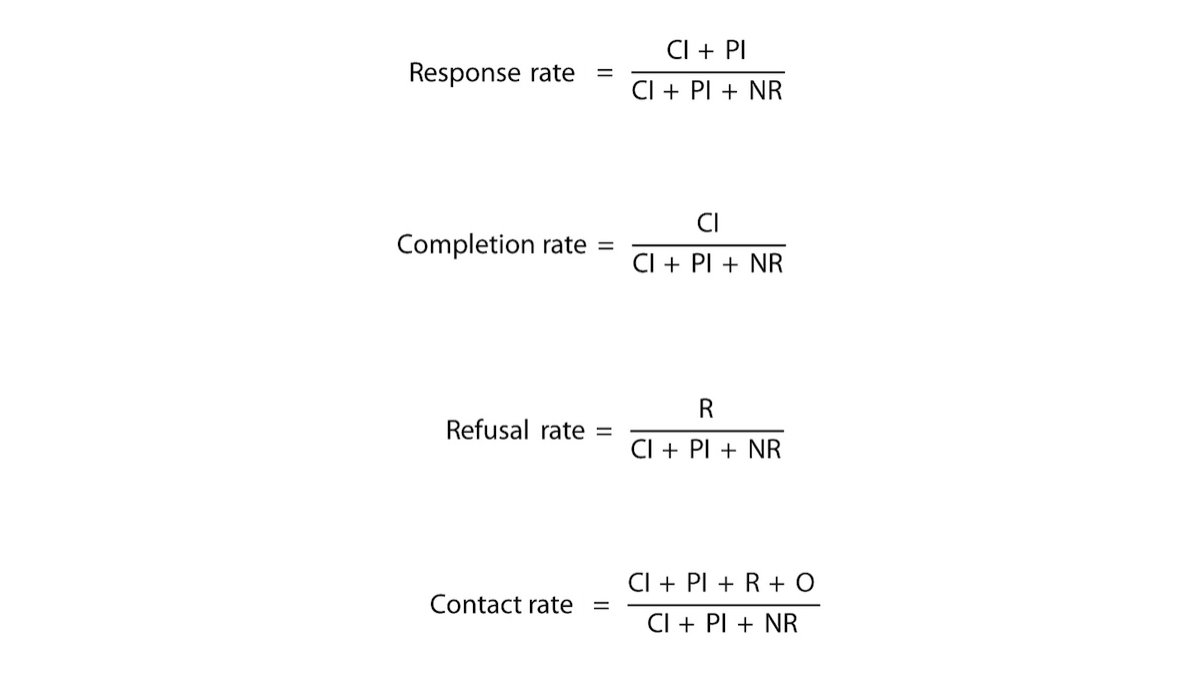
Equations used to calculate the main outcomes.

Complete interviews (CI) are defined as age-eligible respondents who answer 5 or more modules. Partial interviews (PI) are defined as age-eligible respondents who answer between 2 and 4 modules. Participants who are age-eligible but answer less than 2 modules will be classified as a refusal/break-off (R). Non-contacts (NC) are defined as a confirmed number where respondent never picks up (this classification only applies to sub-studies 6 and 7). The main outcomes will be calculated using the equations in Figure 3.

Outcomes will be tabulated and stratified by both the question number and the question content due to the randomization of NCD topic modules. As a secondary analysis, we will conduct Kaplan-Meier curves to plot survey attrition by time spent on survey. For sub-study 7, test for agreement in survey responses between the survey modalities will be assessed using Cohen kappa. Statistical significance will be determined at an alpha of .05.

### Ethical Considerations

The research protocol will receive ethical approval from the institutional review boards of the following institutions: Johns Hopkins University Bloomberg School of Public Health (JHSPH); Makerere University School of Public Health (MakSPH), Uganda; the Uganda National Council for Science and Technology; the Medical Research Coordinating Council (MRCC); the Ifakara Health Institute (IHI), Tanzania; and the Institute of Epidemiology Disease Control and Research (IEDCR), Bangladesh.

## Results

Research activities are expected to be completed in 2017.

## Discussion

National public health surveillance in many LMICs have typically centered on communicable diseases [9]. The emerging burden of NCDs means that many LMICs have to work quickly to set up effective surveillance systems to curb the epidemic. Faced with greater resource constraints than high-income countries, there is need for cost-effective and timely surveillance methods in LMICs. MPSs have the potential to enhance current efforts for systematic collection of NCD risk factor data to identify national trends, as well as to identify segments of the population at particularly high risk for NCDs. If properly harnessed, MPSs have potential to assist LMIC policy makers with resource allocation and choice of NCD prevention and control interventions, among other decisions [15].

The systematic examination of selected incentive characteristics has two potential benefits. First, the optimization of incentive structure and amounts will increase the survey’s contact, response, and completion rates; thereby reducing the number of calls needed to achieve the desired sample size. Second, and only if incentives do not have a differential effect on sub-groups of respondents (ie, incentives increase response in those with more education or of higher socio-economic status), higher response and completion rates may potentially reduce non-response bias.

### Conclusion

The studies discussed in this protocol will evaluate the impact of different strategies to improve IVR survey response and completion rates and to identify the combinations of incentive timing and structure, introduction characteristics, and sampling frames that provide highest yield. Though this protocol centers on NCD risk factors, our findings could potentially be used to inform surveys using IVR methodology for other conditions or aspects such as governance and public opinion.

## Abbreviations

BD4HI: Bloomberg Philanthropies Data for Health Initiative
CATI: computer-assisted telephone interview
FGD: focus group discussion
IEDCR: Institute of Epidemiology, Disease Control and Research
IHI: Ifakara Health Institute
IVR: interactive voice response
KII: key informant interview
JHSPH: Johns Hopkins Bloomberg School of Public Health
LMICs: low- and middle-income countries
MakSPH: Makerere University School of Public Health
MNO: mobile network operator
MPS: mobile phone survey
MRCC: Medical Research Coordinating Council
NCD: non-communicable disease
RDD: random digit dialing
SMS: short message service
